# Peptidome analysis reveals critical roles for peptides in a rat model of intestinal ischemia/reperfusion injury

**DOI:** 10.18632/aging.205200

**Published:** 2023-11-10

**Authors:** Jiaxuan Zhang, Xiaoqi Jiang, Yang Yang, Lei Yang, Bing Lu, Yannan Ji, Leijun Guo, Fan Zhang, Jianhua Xue, Xiaofei Zhi

**Affiliations:** 1 Department of Trauma Center, Affiliated Hospital of Nantong University, Nantong 226001, China; 2 Department of General Surgery, Affiliated Hospital of Nantong University, Nantong 226001, China; 3 Department of Pediatric Surgery, Affiliated Hospital of Nantong University, Medical School of Nantong University, Nantong 226001, China; 4 Department of Clinical Biobank and Institute of Oncology, Affiliated Hospital of Nantong University, Nantong 226001, China; 5 Department of Pediatrics, Affiliated Maternity and Child Health Care Hospital of Nantong University, Nantong 226001, China; 6 Department of Anesthesiology, Affiliated Hospital of Nantong University, Nantong 226001, China

**Keywords:** intestinal ischemia/reperfusion injury, intestinal tissues, peptidomics, LC-MS/MS, peptide Actg2-6

## Abstract

Intestinal ischemia/reperfusion injury (IIRI) has the potential to be life threatening and is associated with significant morbidity and serious damage to distant sites in the body on account of disruption of the intestinal mucosal barrier. In the present study, we have explored this line of research by comparing and identifying peptides that originated from the intestinal segments of IIRI model rats by using liquid chromatography-mass spectrometry (LC-MS). We also analyzed the basic characteristics, cleavage patterns, and functional domains of differentially expressed peptides (DEPs) between the IIRI model rats and control (sham-operated) rats and identified bioactive peptides that are potentially associated with ischemia reperfusion injury. We also performed bioinformatics analyses in order to identify the biological roles of the DEPs based on their precursor proteins. Enrichment analysis demonstrated the role of several DEPs in impairment of the intestinal mucosal barrier caused by IIRI. Based on the results of comprehensive ingenuity pathway analysis, we identified the DEPs that were significantly correlated with IIRI. We identified a candidate precursor protein (Actg2) and seven of its peptides, and we found that Actg2-6 had a more significant difference in its expression, a longer half-life, and better lipophilicity, hydrophobicity, and stability than the other candidate Actg2 peptides examined. Furthermore, we observed that Actg2-6 might play critical roles in the protection of the intestinal mucosal barrier during IIRI. In summary, our study provides a better understanding of the peptidomics profile of IIRI, and the results indicate that Actg2-6 could be a useful target in the treatment of IIRI.

## INTRODUCTION

Intestinal ischemia/reperfusion injury (IIRI) refers to the phenomenon that the intestinal damage is further exacerbated after blood flow is restored [1, 2]. The intestine is one of the most severely affected organs after ischemia/reperfusion (I/R) changes [3]. IIRI is unavoidable in cases of abdominal trauma, hemorrhagic shock, mesenteric vascular ischemic disease, infection, and intestinal transplantation [4, 5]. It can destroy the barrier function of the intestine and lead to multifactorial pathological processes related to bacterial translocation, cell apoptosis, and excessive generation and release of inflammatory cytokines and reactive oxygen species, which can lead to systemic inflammatory response syndrome (SIRS), multiple organ dysfunction syndrome (MODS), and even death [6–8]. As there are very few typical clinical manifestations and early diagnostic markers, it is difficult to diagnose intestinal ischemia in its early phase, and there are no specific and recognized effective methods for treatment of IIRI. Thus, a deeper understanding of its physiopathologic mechanisms can help in the development of new diagnostic methods and treatment strategies.

Peptidomics, a new branch of proteomics, has been widespread concern in the recent few years [9]. Peptidomics focused predominantly on small molecule peptides with relative molecular weights generally less than 10,000 Da, which makes up for the deficiency in traditional proteomic techniques including low sensitivity and low sequence coverage led to a low percentage of identification of small molecules [10]. Peptiomics offers many advantages over proteomics, such as simple structure, convenient operation, extensive research and stable properties [11]. Peptidomics involves quantitative and qualitative analyses of peptides through evolving purification and mass spectrometry methods, and plenty of peptides originating from mammalian cells and tissues have been identified through these methods [12, 13]. Through a series of complex formation and degradation processes, endogenous proteases can guide precursor proteins to split into some functional protein fragments that are called peptides [14]. Peptides are a category of biologically active substances that have been demonstrated to be associated with energy metabolism, DNA damage, cell differentiation, cell protection, and various disease pathways [9, 15]. Peptidomics may be useful for studying endogenous peptides related to IIRI. However, although proteomics methods have been widely applied in the study of IIRI, to the best of our knowledge, no study as yet has applied peptidomics methods for understanding the molecular basis of IIRI. Thus, there is a significant need to utilize peptidomics for the study of IIRI.

Here, we constructed a rat model of IIRI, and compared and identified differentially expressed peptides (DEPs) from the intestinal segments of rats of sham-operated and I/R groups using by liquid chromatography-mass spectrometry (LC-MS). Further, bioinformatics analysis was used to characterize the DEPs and explore potential bioactive peptides related to IIRI. The biological effects of a candidate peptide on the intestinal mucosal barrier during IIRI were also determined. These results may help elucidate the mechanisms underlying IIRI from a peptidomics perspective and provide us with a better understanding of IIRI and, potentially, target peptides for its treatment.

## MATERIALS AND METHODS

### Animal experiments and sample preparation

Adult male Sprague–Dawley rats (weight, 250–280 g) were provided by the Experimental Animal Center of NTU. Rats were maintained according to standard protocols, as described previously [16], and were randomly divided into a control group and experimental groups comprising six rats each. In brief, in the IIRI model rats, pentobarbital (40 mg/kg) was injected intraperitoneally to induce anesthesia, and then atraumatic clips were employed to interrupt the flow of the superior mesenteric artery (SMA) [17]. A sham operation was conducted on six rats to create a control group. In the sham operation group, only laparotomy was performed (with anesthesia induced by the same methods as that used in the model group), and the abdominal incision was closed afterwards. A microbulldog clamp was employed to occlude the SMA for 1 h, and then reperfusion was achieved by loosening the clamps. Reperfusion was performed for 3 h, 6 h, 12 h, 24 h, and 48 h. Samples (1 cm) of ileum tissue present 10 cm away from the ileocecal valve were collected, and then the rats were sacrificed [18, 19]. The resected tissue samples were frozen in liquid nitrogen and maintained at a temperature of -80° C until analysis.

Actg2-6 (50 mg/kg) or an equal volume of sterile water was administered by oral gavage at 4 h prior to surgery in the Actg2-6+I/R group or the I/R group, respectively, followed by 1 h of ischemia and 6 h of reperfusion.

### Hematoxylin-eosin staining

The intestinal tissue samples were fixed in 4% paraformaldehyde, embedded in paraffin, and cut into sections of 6-μm thickness with a microtome. Hematoxylin and eosin (H&E) staining was carried out and evaluated as previously described [20]. Histopathological scores for damage caused to the intestinal tissues were evaluated by two independent pathologists according to Chiu’s method [21].

### Immunohistochemistry staining

Immunohistochemistry (IHC) assay and scoring were conducted as previously described [22]. The formalin-fixed, paraffin-embedded samples were used for the IHC assay, with primary antibody against Ki-67 (Proteintech, China). The immunoreactive scores of the treated intestinal tissues were calculated by two independent pathologists as previously reported [23].

### Peptide extraction and labeling

The peptides of the intestinal tissues were extracted as previously described [24]. All the tissue samples were washed in pre-cooled PBS, and ground into a powder under liquid nitrogen. The powder was transferred to a centrifuge tube, and an appropriate amount of lysis buffer (PBS solution with a final concentration of 1% PMSF) was loaded into the tubes. The mixture was ultrasonically treated on ice for 5 min, and then centrifuged at 4° C for 10 min at 12000 g. The precipitate was discarded, and an equal volume of 100% ACN (acetonitrile) solution was combined with the supernatant and placed on ice. The solution was then centrifuged at 10,000 g for 10 min, and the precipitate was discarded. The supernatant was freeze-dried till it was reduced to half the original volume, and penetrating fluid was collected by centrifugation in a pre-wetted 10-kD ultrafilter tube (UFC501096, Merck Millipore, Germany) at 10000 g for 20 min at 4° C, with 1% trifluoroacetic acid (TFA) added to adjust the pH to 2-3. The Strata X C18 column from Phenomenex (Torrance, CA, USA) was used to desalt the filtrates, and in the next step, a vacuum concentrator was used to dry them. A solution of 0.5 M TEAB was used to dissolve the extracted peptides which were then labelled with the iTRAQ Reagent-8 plex Multiplex Kit based on the manufacturer’s recommendations. After the samples were labeled and mixed, they were separated with the Pierce High-pH Reversed-phase Peptide Fractionation Kit (Thermo Fisher Scientific, Waltham, MA, USA). These samples were divided into 12 fractions and then desalted and vacuum dried on the Strata-X column.

### LC-MS/MS analysis

We conducted peptide identification using the Triple TOF 5600 + LC-MS system (SCIEX, Framingham, MA, USA). The peptide samples were dissolved in a solution containing 2% acetonitrile and 0.1% formic acid, and a TripleTOF 5,600 plus mass spectrometer coupled to an Eksigent NanoLC System (SCIEX, Framingham, MA, USA) was used to evaluate them. The peptide solutions were loaded onto a C18 trap column (5 μm, 100 μm × 20 mm) and then subjected to gradient elution onto a C18 analytical column (3 μm, 75 μm × 150 mm), with the temporal gradient and velocity of flow set to 90 min and 300 nL/min, respectively. One mobile phase was buffer A (2% acetonitrile/0.1% formic acid/98% H_2_O), and another mobile phase was buffer B (98 % acetonitrile/0.1 % formic acid/2 % H_2_O). Information-dependent acquisition (IDA) was applied for MS/MS data acquisition. A primary mass spectrum was scanned with an ion accumulation time of 250 ms, and a secondary mass spectrum comprising 30 precursor ions was acquired with an ion accumulation time of 50 ms. The MS1 spectrum was acquired in the range of 350-1500m/z, and the MS2 spectrum was acquired in the range of 100-1500m/z. The dynamic elimination time of the precursor ions was set to 15 s.

This experiment adopts the fundamental workflow of proteome identification by mass spectrometry, based on which the MS/MS mass spectrometry data and the database were compared and scored after an optimization processing series in order to identify proteins. This method is the most widely used and recognized high-throughput protein identification method in the industry, which has the advantages of high identification accuracy, large flux and no need for manual sequence analysis. Because of the consideration of all possible decoration types and the addition of automatic fault-tolerant matching function, Proteinfilter can retrieve more results than similar software on the premise of ensuring the reliability of identification results, so we use ProteinpilotTM V4.5 (SCIEX, Redwood City, CA, USA). For the identification results of proteinpilot, we further filtered. For the identified proteins, the results were considered reliable if the unused score was ≥ 1.3 (which corresponds to a reliability level higher than 95%) and at least one unique peptide segment was identified per protein. Each of these conditions had to be met for the proteins to be included in this report. For the identified peptide segment and protein quantification, we use conf ≥ 95 for filtration, which means that the reliability is higher than 95%, and each of these conditions had to be met for the peptide segment to be included in this report.

### Bioinformatics analyses

Using the R/bioconductor software, we conducted principal component analysis (PCA). The basic characteristics of each DEP were analyzed with the ProtParam tool (http://web.expasy.org/protparam). The Pfam (http://pfam.xfam.org/) and UniProt (http://www.uniprot.org/) databases were determined to identify whether the peptide sequence was located in the conservative domain or region of their precursor proteins. The peptidase database MEROPS (http://merops.sanger.ac.uk/) was employed to annotate proteolytic events and evaluate substrate specificity. The Open Targets Platform database (http://www.targetvalidation.org/) was employed to evaluate disease-related precursors. The GO (Gene Ontology) (http://geneontology.org) and KEGG (Kyoto Encyclopedia of Genes and Genomes) (http://www.genome.jp/kegg) pathway analyses were employed to determine the potential biological functions and signaling pathways of the peptide precursors, respectively. Using the STRING database, we also conducted a protein-protein interaction (PPI) analysis. In addition, DEPs and their precursor proteins were analyzed with the Ingenuity Pathway Analysis (IPA) software (Qiagen, Redwood City, CA, USA). Finally, the online tool NetWheels (http://lbqp.unb.br/NetWheels/) was employed to generate a helical wheel distribution of candidate peptides.

### Parallel reaction monitoring assay

The parallel reaction monitoring (PRM) assay was employed to detect differences in the abundances of the peptides identified in the label-free peptidomics study, as previously reported [25]. We used the nano UPLC liquid phase system (EASY-nLC1200) for the separation of peptides, and the online Q-Exactive mass spectrometer for their detection. To ensure data quality, the iRT standard peptides (Biognosys, Switzerland) were added to the sample for analysis based on the manufacturer’s instructions. The Skyline 3.6 software was used to analyze the PRM data.

### Peptide synthesis

The amino acid sequence of Actg2-6, a candidate peptide that was identified, is GVMVGMGQKDSYVG. GenScript Biotech (Piscataway, NJ, USA) chemically synthesized Actg2-6 and fluorescein isothiocyanate-labelled Actg2-6 (FITC-Actg2-6) with >95% purity. The procedures were as follows: dissolve the peptide in sterile water and dilute it to the specified concentration prior to use.

### Cell culture and treatment

Caco-2 cells were purchased from the Chinese Academy of Sciences. The recommended conditions, including DMEM medium (Corning Inc., Corning, NY, USA) containing 10% fetal bovine serum (Gibco, Langley, OK, USA) and 1% penicillin/streptomycin (Biyuntian, China), were used. Caco-2 cells were allowed to grow to full confluence and fully differentiate for 14 days, with the medium refreshed every other day.

Caco-2 cells that had reached confluence were incubated with 50 μM Actg2 at 37° C, for 1 h, in the dark, and the cell-penetrating ability of Actg2 was imaged and observed. The cells were allocated to one of the following six groups: (I) control group, (II) Actg2 (50 μM) treatment, (III) H/R + control group, (IV) H/R + Actg2 (10 μM) co-treatment, (V) H/R + Actg2 (20 μM) co-treatment, and (VI) H/R + Actg2 (50 μM) co-treatment. The Caco-2 cell lines were cultured under microaerophilic conditions (Thermo Fisher Scientific, Waltham, MA, USA) containing 1% O_2_, 94% N_2_, and 5% CO_2_ for 12 h then cultured in normoxic condition followed by reoxygenation for 12 h to simulate hypoxia.

### Transepithelial electrical resistance (TEER) assay

The transepithelial electrical resistance (TEER) assay using the Millicell electrical resistance system was employed to monitor the formation of a monolayer of Caco-2 cells, based on a previously reported method [26]. The treated cells were cultured when they were in a stable monolayer state, and the resistance values were monitored daily. The TEER value was calculated as follows and expressed in Ω cm^2^: TEER = (R1 − R0) × A. In the equation, R1 stands for background resistance, R0 represents the collagen layer and membrane insert resistance, and A is the insert membrane area.

### Intestinal permeability assay

FD4 permeability assay (4.4kDa fluorescein isothiocyanate-dextran, Sigma-Aldrich, St. Louis, MO, USA) was used to detect the role of the candidate peptides in intestinal barrier permeability as previously reported with modification [27]. After anoxia/reoxygenation treatment, the top compartment of the monolayers was mixed with FD4 (final concentration of 1mg/mL). The fluorescent intensity in the basal compartment was determined by SN209941 microplate reader (BioTek, Winooski, VT, USA).

### Western blotting

Total protein was isolated from cultured cells and used for Western blotting analysis were carried out as previously reported [28]. Antibodies against Occludin (Santa Cruz Biotechnology, Santa Cruz, CA, USA), ZO-1 (Santa Cruz Biotechnology), and β-actin (Proteintech) were used. β-actin was employed as a loading control.

### Statistical analysis

The data were analyzed by GraphPad Prism 7. The two-tailed Students’ t-test was applied to analyze the DEPs, and significantly different expression was defined at a *P*-value of < 0.01 and fold change value of > 2. Variance analysis was used to examine differences in the size of the groups. Student’s t-test and one-way ANOVA were used to compare differences in variables between groups. Statistical results were presented as mean ± SD. Each experiment was run three times. The results are presented as mean ± SD, and *P < 0.05 was to indicate statistical significance.

## RESULTS

### Peptidomics analysis of rat intestinal tissue

**Figure 1:**
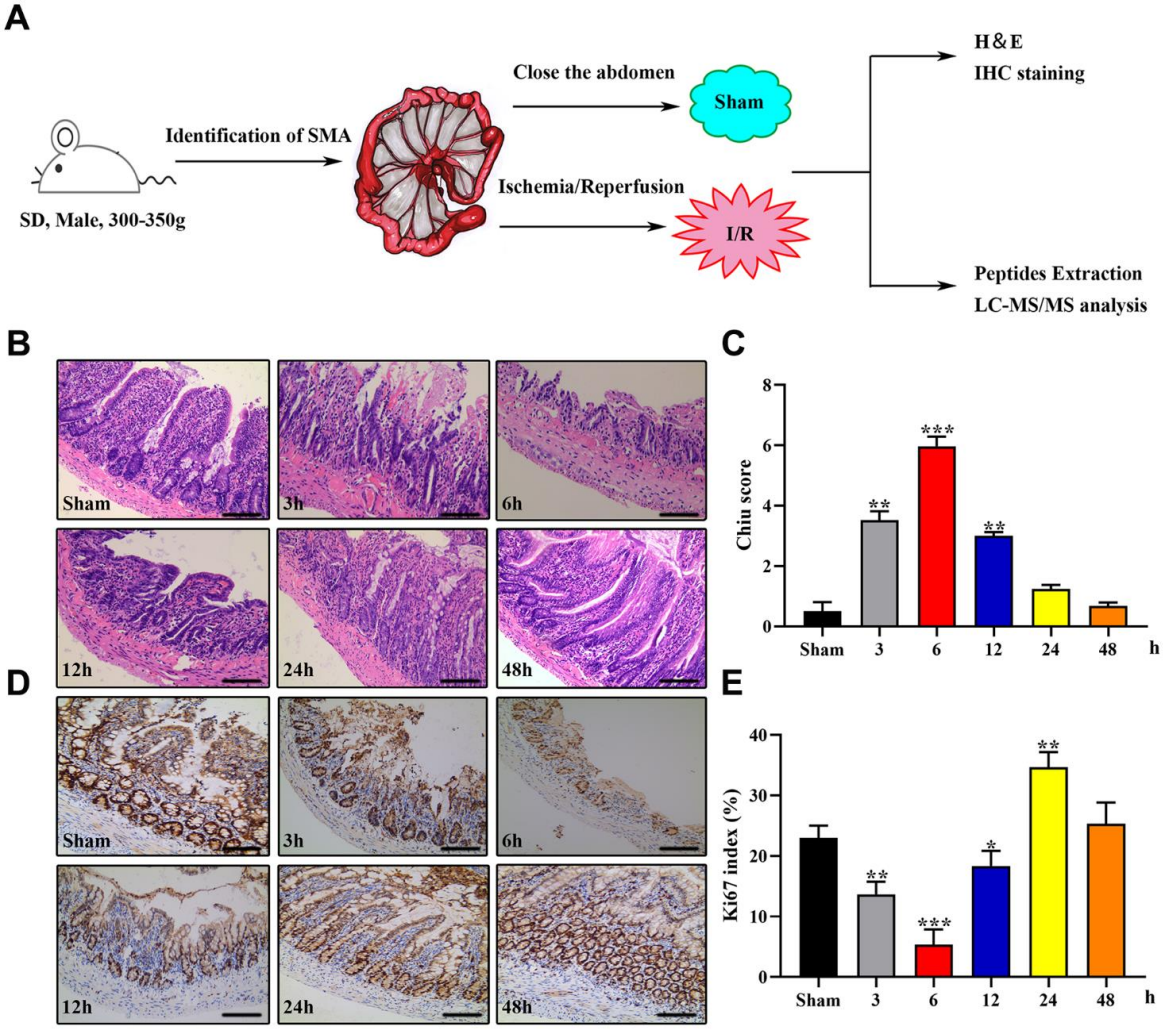
**Establishment of the rat IIRI model.** (**A**) Schematic diagram of the experimental design. (**B**–**E**) Representative images of intestinal sections from rats of the sham operated and I/R groups stained with H&E (**B**) or the IHC marker Ki67 (**C**), respectively, and quantified histopathologically based on Chiu’s score (**D**) or immunoreactive scores (**E**), respectively. *P < 0.05; **P < 0.01; ***P < 0.001.

We used male Sprague–Dawley rats to establish an animal model of IIRI and collected the intestinal tissues for histopathology and LC-MS/MS analysis. A schematic of the protocol is presented in Figure 1A. The most noticeable changes in term of intestinal injury were observed in the 1I/6R (6 h of reperfusion after 1 h of ischemia) group, as evidenced by a higher Chiu score and a lower Ki-67 index (Figure 1B–1E). Therefore, we selected 1I/6R as an optimal time point to perform the peptidomics analysis.

### Identification of DEPs following IIRI

**Figure 2:**
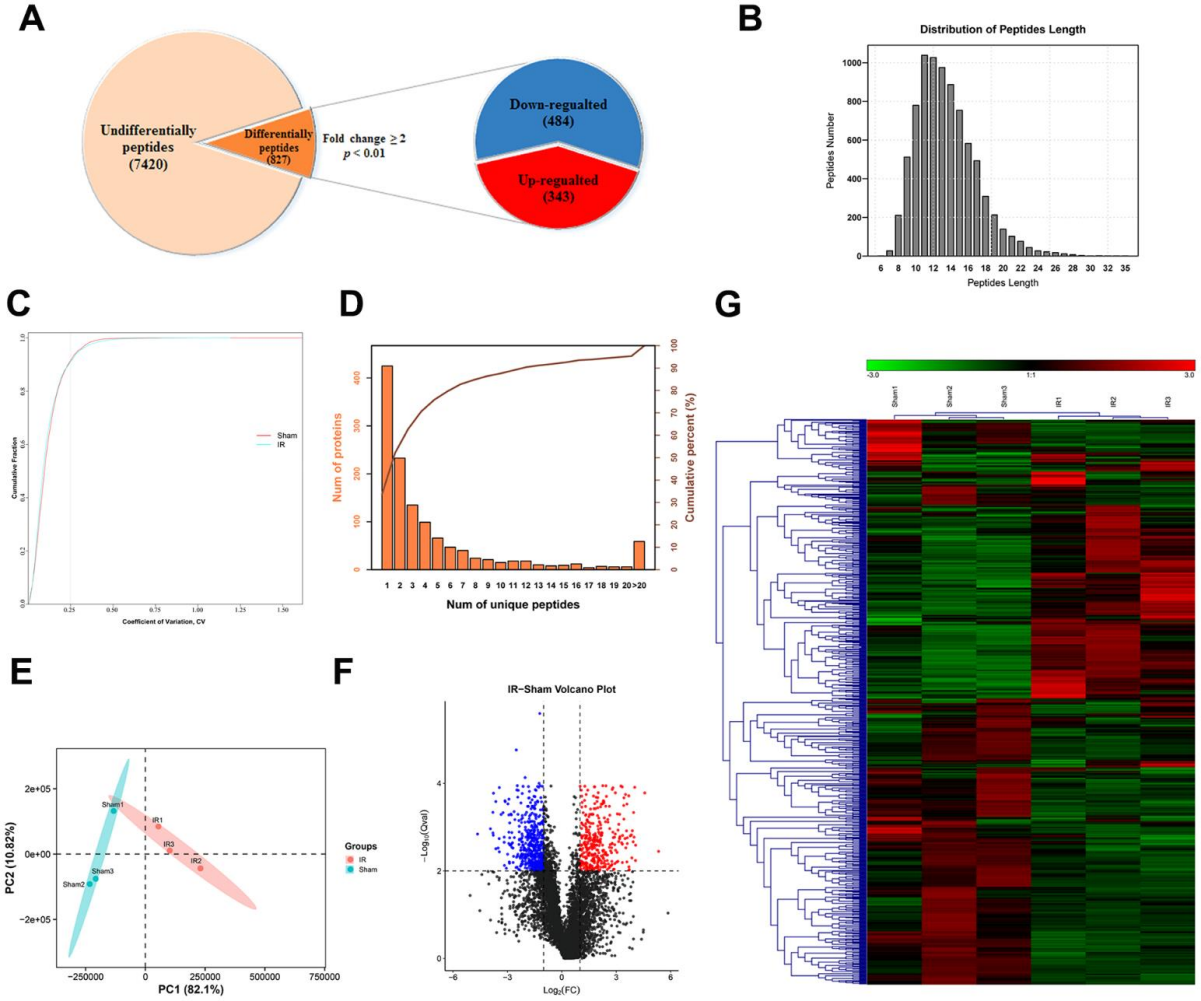
**General characteristic of DEPs identified by LC-MS/MS.** (**A**) Among the 8246 peptides screened, 827 DEPs were identified (fold change ≥ 2 and *p* < 0.01), included 343 upregulated and 484 downregulated peptides. (**B**) Peptide length distribution of DEPs. (**C**) Coefficient of variation of the sham operated and the I/R groups. (**D**) Number of unique peptide lengths. (**E**) Principal component analysis of the peptides identified in each tissue. (**F**, **G**) Volcano plot (**F**) and hierarchical clustering (**G**) of DEPs. The rows represent the expression profiles of DEPs, and the columns represent the corresponding tissue sample. The color red represents higher expression levels, whereas the color blue represents lower expression levels.

The peptide content of intestinal tissue samples from the two groups (sham operation and I/R groups) was examined with LC-MS/MS. A total of 8246 peptides originating from 1,262 precursor proteins were detected. Based on the criteria for significant difference in expression, that is, *P* < 0.01 and fold change ≥ 2 (Figure 2A), 827 DEPs were identified. The average length of the identified DEPs was 13.53, which was within a reasonable range, and the peptides with a length of 11 had the largest number (Figure 2B). Repetitive analysis suggested that the results for both groups were reproducible (Figure 2C). As shown in Figure 2D, 837 precursor proteins contained at least two unique peptides, and this corresponded to 66.32% of the total precursor proteins. Complete separation of the peptide profiles of the two groups was achieved with Principal Component Analysis (PCA) (Figure 2E). Data visualization was accomplished using heatmap and volcano plot of the 827 peptides for normalization and cluster analysis (Figure 2F, 2G). We have listed the 40 DEPs (20 up-regulated and 20 down-regulated DEPs) with the highest fold changes inexpression in Table 1.

**Table 1:** DEPs in the model of IIRI.

**Accession**	**Gene**	**Peptide**	**MW(KDa)**	**Fold change**	***p*-value**
**Up-regulated peptides**					
P63269	Actg2	VITIGNERF	1352	12.197	0.0001557
A0A0H2UHM7	LOC100909441	EPTVIDEVRTGTY	1783	13.684	7.71E-05
Q4JEI8	Defa6	DEDQDVSVSF	1444	13.062	0.0016019
P85108	Tubb2a	IDPTGSYHGDSDLQLE	2050	12.030	0.00077955
P06302	Ptma	DGDEDEEAEAPTGKRVAEDDE	2884	12.057	3.67E-06
A0A0G2K6S9	Myh11	AQKGQLSDDEKF	2015	15.550	8.32E-06
C0JPT7	Flna	KVEYTPYEEGVHSVD	2359	12.187	9.37E-05
Q4QQV0	Tubb6	NATLSVHQLVENTDETY	2238	12.938	1.20E-06
A0A0G2JTV2	Cald1	SVPDEESKPATANAQVEG	2436	13.035	0.00122112
O35413	Sorbs2	TSPGRADLPGSSSTFTT	1985	13.191	0.00155698
D3ZX87	*N/A*	KNLQTVNVDEN	1881	14.296	6.84E-05
A0A0G2JSV6	Hba-a2	FAAFPTTKTY	1754	14.558	0.00056143
P63269	Actg2	QPSFIGMESAGIHETTY	2187	14.849	4.62E-05
P11980	Pkm	PKPDSEAGTAFIQTQQL	2438	15.3097	0.00013413
D3ZHA0	Flnc	IVDPNVDEHSVM	1674	15.8012	8.61E-05
D3ZHA0	Flnc	IVDPNVDEHSVMTY	1938	16.201	3.95E-07
A0A0H2UHM7	LOC100909441	SDKTIGGGDDSFNTF	2168	16.733	0.00068066
D3ZYS7	G3bp1	DVAPAQEDLRTF	1665	16.810	0.00018704
Q4JEI2	Defal1	DPIQEAEEETKTEEQPADEDQDVSVSF	3673	23.803	8.75E-07
G3V7C6	Tubb4b	VPSPKVSDTVVEPY	2124	40.356	0.00031686
**Down-regulated peptides**					
P63269	Actg2	VFPSIVGR	1275	0.040	6.41E-05
P63269	Actg2	MQKEITALAPSTMK	2460	0.065	3.86E-05
F1M853	Rrbp1	TLQEQLENGPNTQLA	1959	0.072	8.47E-06
P63269	Actg2	GILTLKYPIEHG	1962	0.072	5.68E-05
Q5BJ93	Eno1	GDDLTVTNPK	1667	0.073	7.15E-05
Q10758	Krt8	LNPLKLEVDPNIQAV	2270	0.073	9.42E-07
P85834	Tufm	GTVVTGTLER	1336	0.080	2.83E-06
P63039	Hspd1	SIVPALEIANAHR	1695	0.080	0.00025387
P10111	Ppia	EGMSIVEAMERFGS	1862	0.088	0.0002383
Q9QXQ0	Actn4	ALDFIASK	1472	0.091	0.00010306
P63039	Hspd1	QSKPVTTPEEIAQ	2035	0.092	1.60E-07
A0A0G2KAJ7	Col12a1	ITYQPSTGEGNEQTTTVGGR	2399	0.092	0.00032739
Q10758	Krt8	KLEVDPNIQAV	1833	0.098	1.01E-05
A0A0H2UHM5	Pdia3	TADGIVSHL	1216	0.106	8.29E-06
B0K010	Txndc17	ITAVPTLLK	1563	0.111	0.00014108
P34058	Hsp90ab1	KHLEINPDHPIVETLR	2518	0.111	8.69E-05
P63269	Actg2	GVMVGMGQKDSYVG	2051	0.112	0.00107894
A0A0H2UHM7	LOC100909441	YAPVISAEK	1585	0.114	2.36E-06
Q10758	Krt8	KLEVDPNIQA	1734	0.115	2.81E-06
P63269	Actg2	GYSFVTTAEREIV	1775	0.120	0.00139115

### Characteristics of the identified DEPs

**Figure 3:**
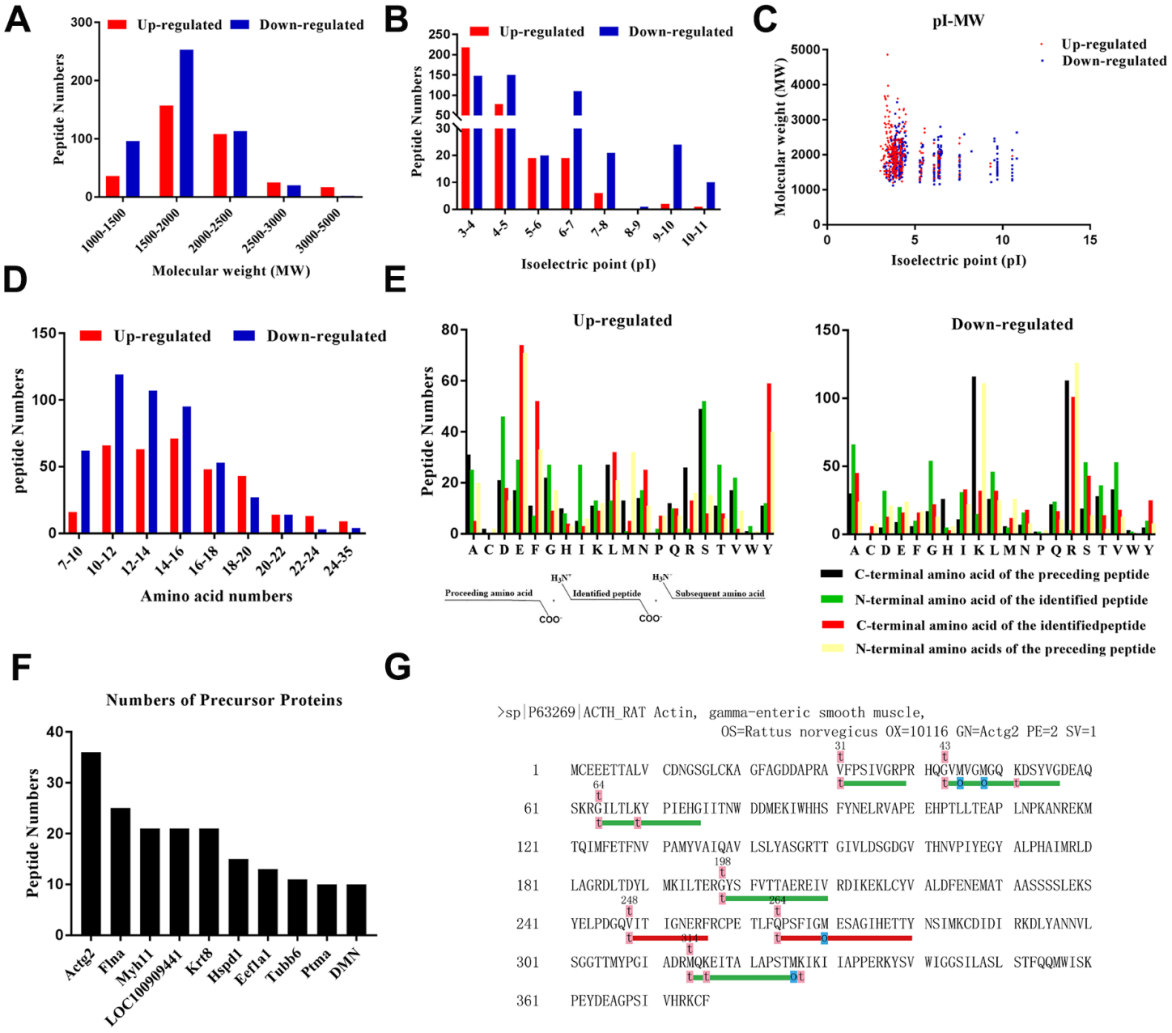
**Characteristics of the identified DEPs.** (**A**) Molecular weight distribution. (**B**) Isoelectric point distribution. (**C**) Scatter plot of molecular weight versus isoelectric point. (**D**) Length in terms of the number of amino acids. (**E**) Distribution of the cleavage sites of the DEPs and diagram of the cleavage site distribution. (**F**) Peptides sharing the same precursors. (**G**) Peptides that originated from the precursor Actg2.

The general characteristics of the DEPs were evaluated. The results indicated that the molecular weight (MW) of most of the peptides was within a broad range of 1,000 to 2,500 Da (Figure 3A), and their isoelectric point (pI) was in the range of 3.0 to 7.0 (Figure 3B). Next, we evaluated the scatterplot of MW/pI of the DEPs (Figure 3C). The number of amino acids within the DEPs ranged from 9 to 99 (Figure 3D). Interestingly, several peptides originated from a single parent protein. Figure 3F depicts the top ten identified peptides. Actg2 possessed the highest number of the identified DEPS in comparison to the other precursor proteins. Peptides are released from precursor proteins in specific tissues based on the nature of the cleavage enzymes involved, and thus, peptide levels are regulated by these enzymes [29]. We integrated the LC-MS/MS results with bioinformatics data to determine the specificity of the cleavage sites at the amino terminus (N-terminus) and carboxyl terminus (C-terminus) of the peptides (Figure 3E). We observed that serine (S), serine (S), glutamic acid (E), and glutamic acid (E) were the four dominant cleavage sites in the up-regulated peptides, whereas lysine (K), alanine (A), arginine (R), and arginine (R) constituted the four main cleavage sites in the down-regulated peptides. Furthermore, we attempted to build a “peptide alignment map” by aligning the peptide sequences against the sequence of the corresponding precursor protein (Figure 3G). Based on our sequencing results, Actg2 contributed to the largest number of identified peptides, which were easily and selectively cleaved by certain types of enzymes, and they might have bioactive effects.

### Identification of bioactive peptides potentially associated with ischemia reperfusion injury

We screened the domain information to examine and compare specific domain structures or patterns of precursor proteins in relation to the DEPs through the Pfam and UniProt database (Table 2). We observed that most of the peptides were mainly located in the functional domains of the precursors, with 15 up-regulated and 19 down-regulated based on our retrieval results. In particular, six peptides derived from the precursors Hsp90ab1, Pdia3, Ppia, Hspd1, and Eno1 and located within their functional domains were found to be tightly associated with ischemia reperfusion injury, based on data from the Open Targets Platform database (Table 3). Encouraged by these results, we further studied the properties of the putative peptides in the intestines of the rats with ischemia reperfusion injury.

**Table 2:** DEPs located in the functional domains of their precursor proteins.

**Peptide sequence**	**Protein**	**Location**	**Domain**	**Description**
**Up-regulated peptides**				
VITIGNERF	Actg2	248-256	3-376	Actin
EPTVIDEVRTGTY	LOC100909441	71-83	48-245	Tubulin
DEDQDVSVSF	Defa6	37-46	1-50	Defensin_propep
IDPTGSYHGDSDLQLE	Tubb2a	30-45	3-212	Tubulin
DGDEDEEAEAPTGKRVAEDDE	Ptma	76-96	42-101	Asp/Glu-rich (acidic)
KVEYTPYEEGVHSVD	Flna	1318-1332	1250-1349	Filamin
NATLSVHQLVENTDETY	Tubb6	184-200	47 – 244	Tubulin
SVPDEESKPATANAQVEG	Cald1	144-161	102-623	Caldesmon
FAAFPTTKTY	Hba-a2	34-43	3 – 142	Globin
QPSFIGMESAGIHETTY	Actg2	264-280	3-376	Actin
IVDPNVDEHSVM	Flnc	241-252	160 – 263	Calponin-homology (CH) 2
IVDPNVDEHSVMTY	Flnc	241-254	160 – 263	Calponin-homology (CH) 2
SDKTIGGGDDSFNTF	LOC100909441	38-41	2-213	Tubulin
DPIQEAEEETKTEEQPADEDQDVSVSF	Defal1	20-46	1-51	Defensin_propep
VPSPKVSDTVVEPY	Tubb4b	273-286	223 – 347	Tubulin
**Down-regulated peptides**				
VFPSIVGRP	Actg2	31-39	3-376	Actin
MQKEITALAPSTMK	Actg2	314-327	3-376	Actin
GILTLKYPIEHG	Actg2	64-75	3-376	Actin
GDDLTVTNPK	Eno1	317-326	142 – 431	Enolase_C
LNPLKLEVDPNIQAV	Krt8	73-87	2-87	Keratin_2_head
GTVVTGTLER	Tufm	272-281	272-341	GTP_EFTU_D2
SIVPALEIANAHR	Hspd1	255-268	47-550	Cpn60_TCP1
EGMSIVEAMERFGS	Ppia	134-147	7 – 163	PPIase cyclophilin-type
ALDFIASK	Actn4	115-122	50 – 154	Calponin-homology (CH) 1
QSKPVTTPEEIAQ	Hspd1	158-170	47-550	Cpn60_TCP1
ITYQPSTGEGNEQTTTVGGR	Col12a1	1787-1806	1757 – 1851	Fibronectin type-III
KLEVDPNIQAV	Krt8	77-87	2-87	Keratin_2_head
TADGIVSHL	Pdia3	125-133	31-135	Thioredoxin
ITAVPTLLK	Txndc17	90-98	9 – 122	DUF953
KHLEINPDHPIVETLR	Hsp90ab1	624-639	620 – 723	Interaction with NR1D1
GVMVGMGQKDSYVG	Actg2	43-56	3-376	Actin
YAPVISAEK	A0A0H2UHM7	271-279	247 – 392	Tubulin_C
KLEVDPNIQA	Krt8	77-86	2-87	Keratin_2_head
GYSFVTTAEREIV	Actg2	198-210	3-376	Actin

**Table 3:** Protein precursors and identified peptides related to ischemia reperfusion injury.

**Gene**	**Description**	**Peptide numbers**	**Association score with ischemia reperfusion injury^#^**
Hsp90ab1	Heat shock protein HSP 90-beta	1	0.03
Pdia3	Protein disulfide-isomerase	1	0.21
Ppia	Peptidyl-prolyl cis-trans isomerase A	1	0.02
Hspd1	60 kDa heat shock protein, mitochondrial	2	0.02
Eno1	Enolase 1, (Alpha)	1	0.04

^#^The association score comes from open targets platform database.

### Bioinformatics analysis of DEPs

**Figure 4:**
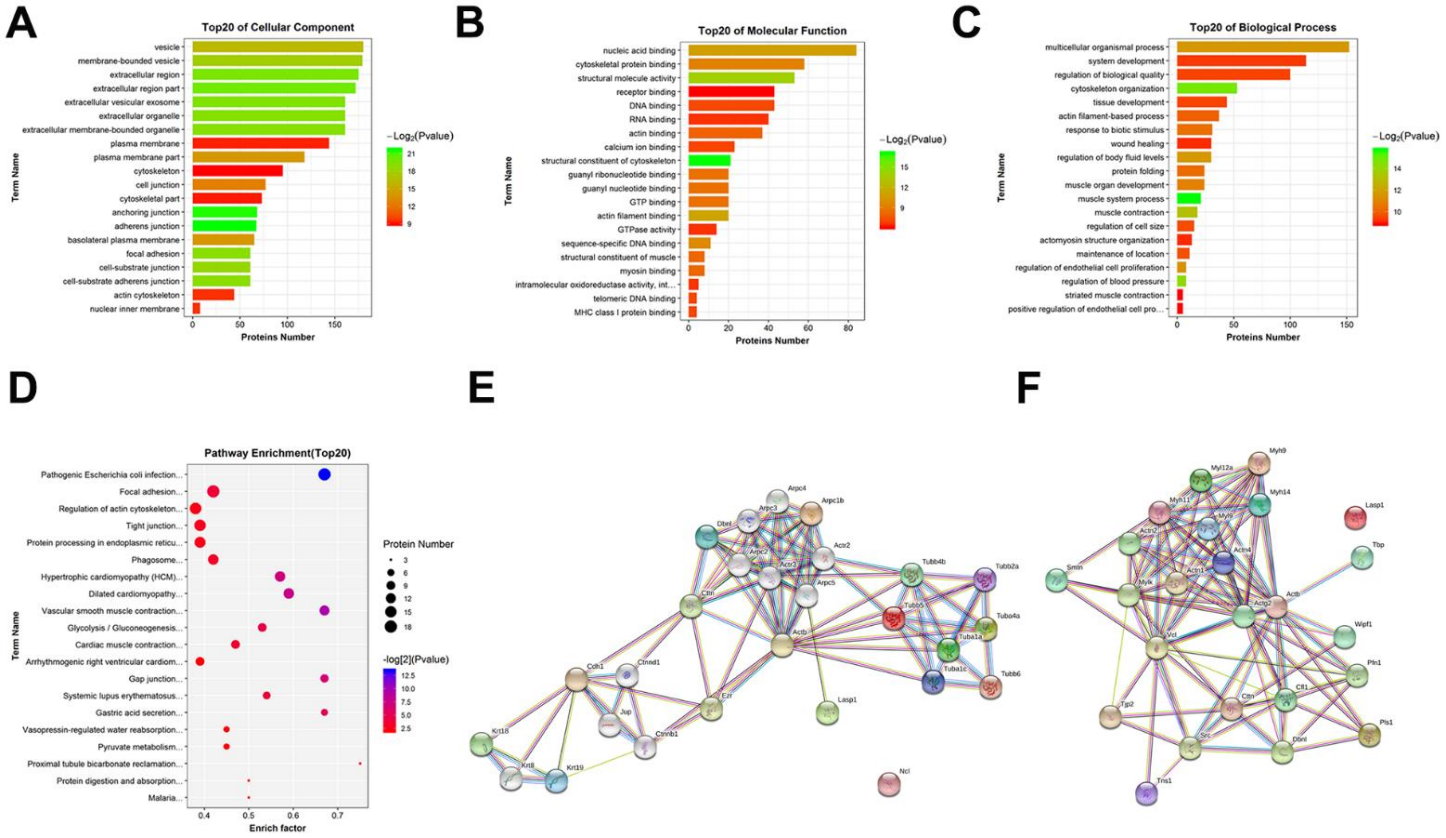
**GO and KEGG pathway analysis of the precursor proteins of DEPs.** (**A**) Cellular components. (**B**) Molecular functions. (**C**) Biological processes. (**D**) KEGG pathway analysis. (**E**) Interaction network analysis of pathogenic *Escherichia coli* infection. (**F**) Interaction network analysis of TJ. These network images were generated by STRING.

**Figure 5:**
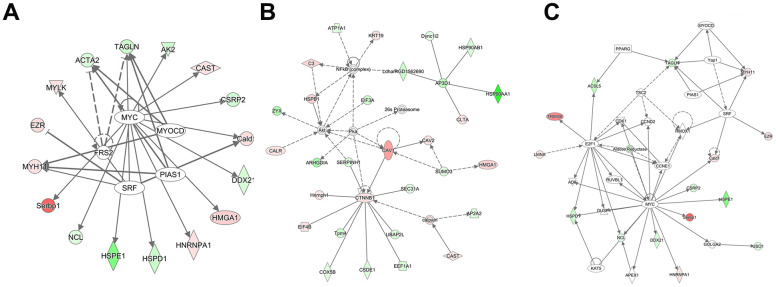
**Analysis of the interaction network and upstream effects of the 827 DEPs and their precursor proteins.** (**A**) Analysis of upstream effects in these biological events. (**B**) Networks related to endocrine system disorders, organismal injury, and abnormalities. (**C**) Networks related to cell cycle, cell death and survival, and gene expression. The intensity of the node color reflects the degree of upregulation (green) or downregulation (red).

To determine whether the identified peptides were associated with IIRI, bioinformatics analyses were employed to determine the potential biological functions of the DEPs by evaluating their precursor proteins. The GO functional annotation is made up of three major components: cellular components, molecular functions, and biological processes. As shown in Figure 4A, with regard to cellular components, vesicle, membrane−bounded vesicle, extracellular region, extracellular region part, extracellular vesicular exosome, extracellular organelle, etc., were the most significantly enriched. For the molecular functions, nucleic acid binding, cytoskeletal protein binding, structural molecule activity, receptor binding, DNA binding, RNA binding, etc., were the most significantly enriched (Figure 4B). As shown in Figure 4C, the most highly enriched biological processes were multicellular organismal process, system development, regulation of biological quality, cytoskeleton organization, tissue development, actin filament-based process, etc. The KEGG pathway results emphasized on the pathways involved in pathogenic *Escherichia coli* infection, focal adhesion, regulation of actin cytoskeleton, tight junction (TJ), and protein processing in endoplasmic reticulum, among others (Figure 4D). Subsequently, data on protein-protein interaction and co-occurrence predicted by KEGG pathway analyses were searched against the STRING database to identify the networks relevant to the precursor proteins. Two of the interaction networks of the parent proteins of the identified DEPs—pathogenic *Escherichia coli* infection and TJ—were represented in Figure 4E, 4F. Additionally, Ingenuity Pathways Analysis (IPA) was used to determine the upstream effects and interaction networks based on peptidomic data. The top-scoring biological networks that were identified as being potentially correlated with IIRI were “endocrine system disorders, organismal injury and abnormalities” and “cell cycle, cell death and survival, gene expression” (Figure 5B, 5C). The possible upstream effects preceding these biological events were also predicted by IPA (Figure 5A).

### Expression profiles and basic features of the candidate peptides

**Figure 6:**
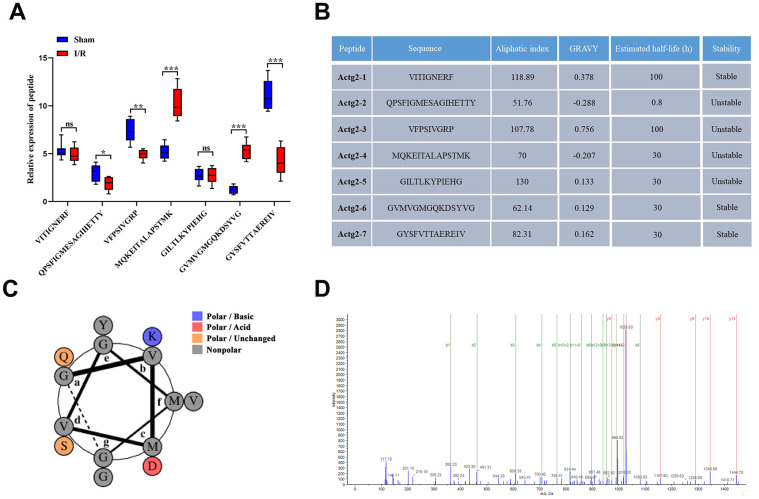
**Expression profiles and basic features of Acgt-2-derived peptides.** (**A**) Relative expression levels of seven peptides derived from Acgt-2 were evaluated by PRM. (**B**) Basic features of Actg-2 derived peptides. (**C**) Helical wheel projections of Actg2-6. (**D**) The product ion spectrum of Actg2-6. ns=not significant, *P < 0.05; **P < 0.01; ***P < 0.001.

From the peptide sequencing data, the maximum number of differentiated peptides (n = 7) were found to originate from Actg2. The seven candidate peptides, namely, VITIGNERF, QPSFIGMESAGIHETTY, VFPSIVGRP, MQKEITALAPSTMK, GILTLKYPIEHG, GVMVGMGQKDSYVG, and GYSFVTTAEREIV, which were derived from the precursor protein Actg2, were termed Actg2-1, Actg2-2, Actg2-3, Actg2-4, Actg2-5, Actg2-6, and Actg2-7, respectively. The seven peptides were analyzed by PRM mass spectrometry, and the results indicated that the expression changes in most of the peptides were in agreement with the results from the peptidomics analyses (Figure 6A). The ProtParam software showed that the aliphatic index of the seven peptides was 118.89, 51.76, 107.78, 70, 130, 62.14, and 82.31, respectively; the grand average of hydropathicity (GRAVY) was 0.378, -0.288, 0.756, -0.207, 0.133, 0.129, and 0.162, respectively; and the estimated half-life was 100, 0.8, 100, 30, 30, 30, and 30 h, respectively. Among the seven peptides, three peptides were predicted to be stable, but four peptides exhibited instability (Figure 6B). Collectively, our results indicated that Actg2-6 had more significant differences in expression, a longer half-life and better properties of lipophilicity, hydrophobicity and stability. According to the helical wheel projections, Actg2-6 possessed one polar/basic residue, one polar/ acid residues, two polar/uncharged residues and ten nonpolar residues (Figure 6C). The peptide spectrum demonstrated the presence of the fragment spectrum of peptide Actg2-6 in Actg2 (Figure 6D).

### Effects of the candidate peptide on the intestinal mucosal barrier

**Figure 7:**
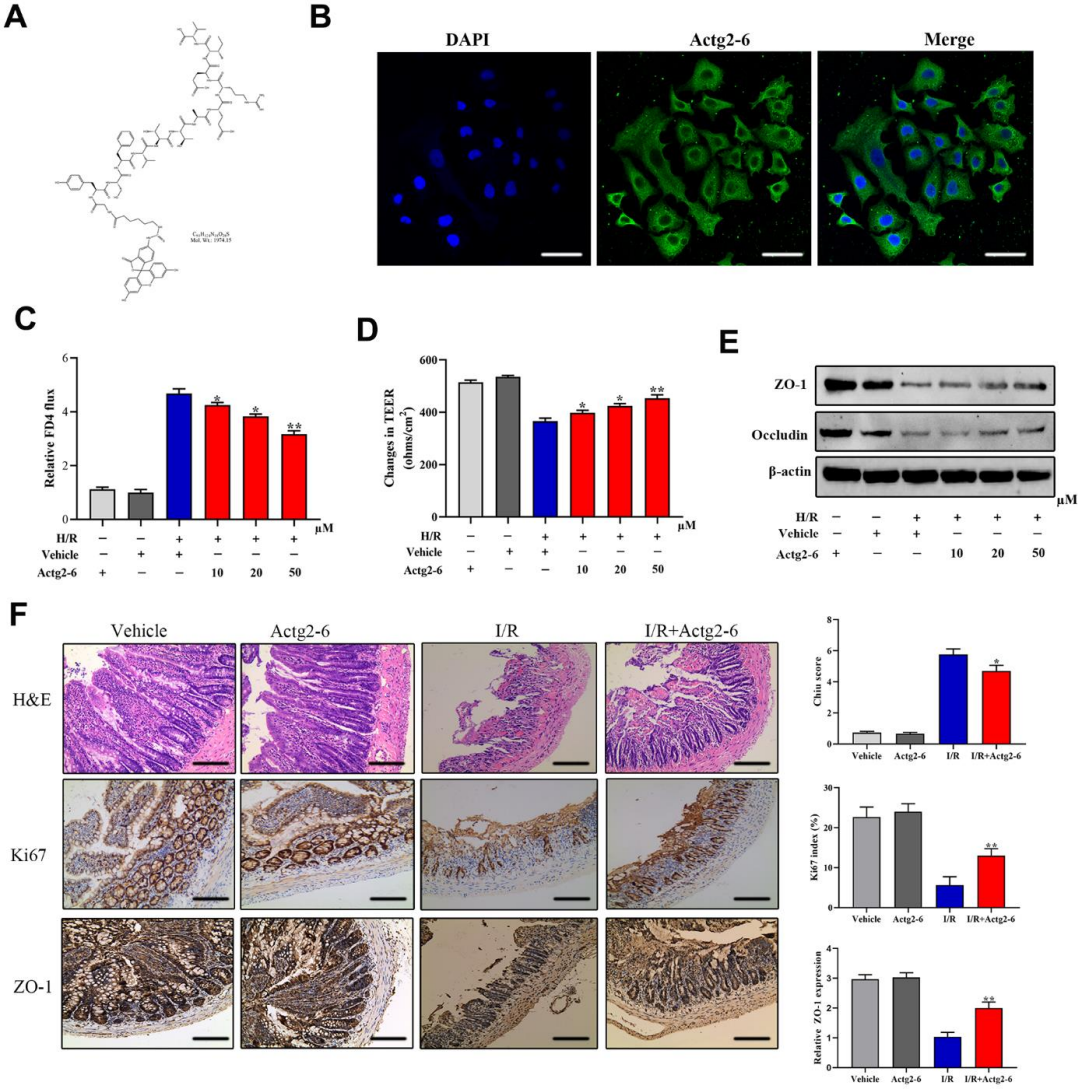
**Effects of the candidate peptide Acg2-6 on intestinal mucosal barrier function.** (**A**) Chemical formulas of Actg2-6. (**B**) Representative images of differentiated Caco-2 cells after incubation with Actg2-6. (**C**) Effect of Actg2-6 on intestinal epithelial permeability, as evaluated by the FITC-dextran paracellular permeability assay. (**D**) Effect of Actg2-6 on intestinal epithelial permeability, as evaluated by the TEER assay. (**E**) The proteins related with TJ proteins were detected by Western blotting. (**F**) Representative images of intestinal sections from rats of the sham operated and 1I/6R groups stained with H&E or the IHC marker Ki67 or ZO-1, respectively, and quantified histopathologically based on Chiu’s score or immunoreactive scores, respectively. *P < 0.05; **P < 0.01; ***P < 0.001.

We investigated the effects of the candidate peptide Actg2-6 on intestinal mucosal barrier function. To this end, differentiated Caco-2 cells were treated with chemically synthesized Actg2-6 (Figure 7A, 7B). As shown in Figure 7C, Actg2-6 exhibited inhibitory effects on intestinal barrier dysfunction, as evidenced by a decrease in the paracellular permeability of FITC-dextran and an increase in the TEER value. TJ proteins are the structural components that regulate paracellular permeability. Thus, the expression of TJ proteins was determined to evaluate the effect of Actg2-6 on intestinal barrier dysfunction. Our findings indicated that Occludin and ZO-1 were markedly upregulated in the Caco-2 cells treated with Actg2-6 (Figure 7D). We next evaluated the effects of Actg2-6 *in vivo*. Compared with the I/R group, treatment of Actg2-6 significantly attenuated the intestinal histological injury and increased intestinal epithelial proliferation (Figure 7F). Consistent with these findings, the protein expression of ZO-1 by IHC assay was higher in the I/R+ Actg2-6 group than in the I/R group (Figure 7F)). These findings indicated that the candidate peptide Actg2-6 might participate in the protection of the intestinal mucosal barrier during IIRI.

## DISCUSSION

The clinical signs and symptoms of IIRI are mostly nonspecific and sometimes make diagnosis difficult; in addition, it is often accompanied by poor prognosis and a reduction in survival [30–32]. IIRI not only results in injury to the small intestinal tissues, but also triggers serious damage in distant sites of the body on account of disruption of the intestinal mucosal barrier that leads to widespread regional and systemic damage [33, 34]. Surgical resection of the small intestine is still the primary treatment modality in most patients with IIRI; however, it often results in bowel problems including short bowel syndrome, that affect the rest of the patients’ life [35]. The reported survival rate of IIRI ranges from 39% to 50% in human and veterinary patients [36]. It is, therefore, imperative to explore novel diagnostic approaches and develop potent interventions for IIRI.

As a widely known ischemic organ, the intestine has been widely studied using comparative and quantitative miRNA profiling and proteomic methods [37–39]. Recent advances in peptidomics, which is an emerging field derived from proteomics are actively being used to study multiple diseases, such as bronchopulmonary dysplasia, acute myocardial infarction, and neonatal respiratory distress syndrome [9, 40, 41]. Some of the advantages of peptide treatment are low molecular weight, low toxicity, and specific targeting, and this makes it a promising new regime that could be applied to a wide range of diseases [29, 42–45]. However, supporting data from peptidomics studies of the intestine are currently unavailable. Therefore, we comprehensively screened and identified peptides in rat intestinal tissues following ischemia/reperfusion and conducted a comparative peptidomics analysis. Compared with human specimens, animal models offer the advantages of controllability of the experimental conditions, which can not only ensure the uniformity of the experimental specimens but also overcome many other limitations of human samples. To the best of our knowledge, our study is the first present data from peptidomics analysis of IIRI. Our work is expected to provide theoretical underpinnings for subsequent studies about IIRI and may offer meaningful approaches to improve the prevention and treatment of IIRI.

In this research, we determined the possible biological activities of peptides that were identified as being associated with IIRI and provided considerable support for any follow-up work. We used 10-kDa MWCO filters to remove surplus proteins from the rat intestines, but this did not affect peptide recovery. We identified a total of 827 DEPs that had originated from 1262 parent proteins. The distribution of unique peptides can help determine the existence of corresponding proteins. Our data indicated that precursor proteins containing at least two unique peptides were abundant and constituted the majority of the total proteins. Unique peptide segments are peptides that exist only in one protein. The presence of this type of peptide segment can uniquely determine the presence of the corresponding protein. In addition, the length of the peptides was within a reasonable range. There was little difference between the sham operated and I/R groups in term of the coefficient of variation, which indicated that the peptidomic analysis was stable and reliable. Furthermore, MW and pI, which are the basic features of these identified peptides, reflected differences in the distribution of the peptides between the two groups, as well as revealed that the peptide extraction method was rather effective. We also found that multiple peptides shared the same precursor protein. That is, catalysis of the same active protein precursor may sometimes result in the production of two or more components with different biological functions.

The PCA plot indicated that there was a certain degree of clustering, but there was obvious separation and little overlap between the two groups (that is, the sham-operated group and I/R group). Thus, their peptidomics profiles exhibited significant differences in terms of various pathophysiological processes. Previous studies have revealed that protease activity can represent the intestine’s functional status, and multiple physiological or pathologically processes have long been closely correlated with alterations in proteolytic systems [9, 46, 47]. Proteases serve as a bridge between proteomics and peptidomics [48]. They catalyze the hydrolysis of peptides, which are a class of smaller fragments of proteins [29], and can be classified into five groups: metalloproteases, cysteine proteases, serine proteases, aspartic acid proteases, and threonine proteases [49]. The specificity and activity of protease cleavage can be determined by their cleavage patterns, but the protease activities of different cleavage sites probably differ [50]. We identified the cleavage sites of the peptides and observed that the frequency at which the cleavage site was at N- or C-terminal varied in different stages. Further, the cleavage of peptides by proteases was based on specific rules and different conditions, as evidenced by a unique series of proteases that were active in the context of IIRI. Site-specific cleavage makes the identification of specific markers possible [51]. Therefore, changes in specific protease functions warrant further investigation.

Sequence of Actg2 and the distribution of identified peptides were shown in Figure 3G. The letter o, highlighted in blue, indicates methionine oxidation, and the letter t, highlighted in pink, indicates N-terminal and lysine acetylation. Met is extremely susceptible to oxidation, which can lead to a modification of the surface hydrophobicity of affected proteins and in a local change of folding, and it can be modified by all types of reactive oxygen species, thereby clarifying the reactive oxygen groups in the body [52, 53]. Acetylation modification has been classified into two main groups: Lysine acetylation and N-terminal acetylation [54]. Lysine acetylation is a dynamic post-translational modification process that is reversible and plays an important role in regulating protein function, chromatin structure, and gene expression [55, 56]. Unlike lysine acetylation, N-terminal acetylation refers to the transfer of an acetyl group to the N terminus of a protein, i.e., the amino group of the first residue in proteins [57]. N-terminal acetylation can promote membrane targeting of certain proteins by interacting with intact membrane proteins or directly binding to membrane lipids [58]. From the above results, it is not difficult to show that these peptides originated from Actg2 all have modifications, and the candidate peptide, Actg2-6, may have better property of lipophilicity, hydrophobicity and stability due to its more modifications.

Protein domains as building blocks of all proteins, are evolutionarily and structurally conserved, as well as have specific functions; moreover, they can change, function, and exist independently of the rest of the protein chain [9, 59, 60]. In this study, the online UniProt and Pfam databases were used to identify more potential bioactive peptides. As illustrated in Table 2, we observed that the identified peptides were mostly located in the functional domains. Additionally, we also identified the precursor proteins associated with ischemia reperfusion injury based on the Open Targets Platform database. These progenitor proteins have previously been reported to play critical roles in ischemia reperfusion injury. For instance, FLNA was found to be mostly enriched in the focal adhesion domain; further, FLNA may have effects on the shape of the intestinal epithelia and cell permeability and, thereby, promote the progress of IIRI [61].

Further, PDIA3 could be promoted by remifentanil and inhibit various IIRI-mediated stresses by activating p38MAPK [62]. Previous studies have shown that Ppia was highly up-regulated in myocardial I/R injury, and mainly interacted with the chaperonin-containing TCP1 complex and Usp47 [63]. Up-regulation of Ppia was considered to be a protective mechanism against adverse ischemia/reperfusion conditions. A search of the Open Targets Platform database revealed five target proteins that may play a biological role in ischemia reperfusion injury. Overall, these observations indicated that the peptides related to IIRI might have potential functions similar to their precursors, and this is worthy of further functional research.

Previous research has demonstrated that peptides often exert similar or opposing biological functions as their precursor protein [60]. Therefore, we used a bioinformatics software to obtain crucial biological information about the parent proteins of the DEPs involved in the IIRI process. Significant structural alterations caused by IIRI that may induce intestinal epithelial programmed cell death and, thereby, disruption of the intestinal epithelial barrier were observed in the intestine [64]. According to the results of GO enrichment analysis, the following terms were enriched: cell components: cell junction, cytoskeleton, anchoring junction, and adherens junction; molecular functions: cytoskeletal protein binding, calcium ion binding, and actin filament binding; biological processes: response to biotic stimulus, regulation of body fluid levels, and wound healing. KEGG analysis revealed that pathogenic *Escherichia coli* infection, TJ, regulation of actin cytoskeleton and focal adhesion had a high enrichment score. The cytoskeleton can maintain the normal structure of intestinal mucosal barrier, and is key to the transportation and functional integrity of all eukaryotic cells, including intestinal epithelial cells [65, 66]. The cell junction plays critical roles in the intestinal mucosal barrier, especially TJ, which can effectively prevent bacteria and endotoxins from being released into the bloodstream through the intestinal mucosa [67, 68]. If the TJ is destroyed, the intestinal permeability will increase and, subsequently, intestinal mucosal barrier function will be damaged [69]. Therefore, the intestinal mucosal barrier is vital for IIRI. In addition, the results of IPA revealed many precursors that might play important roles in networks associated with endocrine system disorders, organismal injury and abnormalities and cell cycle, cell death and survival, and gene expression, and confirm the close association of DEPs with IIRI. Overall, the bioinformatics analysis indicated that the DEPs identified might play vital regulatory roles in intestinal mucosal barrier function following IIRI.

The abovementioned results provide theoretical possibilities for determining the biological effects of DEPs. It is worth noting that one novel peptide was identified in this study, namely, Actg2-6. This candidate peptide was derived from the precursor protein Actg-2 (Actin Gamma 2, Smooth Muscle Actg2). Previous studies have suggested that Actg2 is expressed predominantly in intestinal smooth muscles and actively participates in smooth muscle contractility [70]. There is also evidence to indicate that Actg2 is present on most cell types and is a component of the cytoskeleton and a medium for internal cell motility [71]. Moreover, Actg2 is cleaved posttranslationally to generate shorter peptides after sequential proteolytic cleavages. The results of PRM mass spectrometry and bioinformatics analysis conducted in the present study suggest that Actg2-6 has a more significant expression difference, a longer half-life and better lipophilicity, hydrophobicity, and stability than the other Actg2 peptides identified and, therefore, deserves further in-depth study. We demonstrated that Actg2-6 treatment of Caco-2 cells inhibited the increase in permeability induced by H/R treatment, as confirmed by the increased TEER value and decreased FITC-dextran paracellular permeability. Thus, Actg2-6 might play a role in alleviating the injury to the intestinal mucosal barrier.

Next, we determined the correlation between TJ proteins and Actg2-6. Previously, TJ proteins were demonstrated to be involved in the maintenance of the function of the intestinal mucosal barrier, which regulates paracellular permeability to water, ions, and nutrients [72]. TJ proteins are involved in a dynamic regulation mechanism associated with physiological and pathological conditions that is required for maintaining equilibrium in intestinal permeability [73]. During the process of IIRI, increased permeability of the intestine is accompanied by disruption of the expression and structure of TJ proteins [74]. TJ proteins, particularly ZO-1 and Occludin, are the most important transmembrane proteins involved in maintaining barrier function of the intestinal mucosa [75]. Accordingly, downregulation of Occludin results in an increase in TJ permeability in the intestines in a non-restrictive manner [76, 77]. Further, ZO-1 has been shown to play critical roles as a TJ adaptor protein in the regulation of adherens junctions and the transportation of ions and macromolecules between cells such as endothelial and epithelial cells [78, 79].

Based on these findings, we confirmed that enhancement of intestinal mucosal barrier function promoted by Actg2-6 treatment was correlated with upregulation of ZO-1 and Occludin. We found that Actg2-6 also had significant biological effects *in vivo*, reducing intestinal tissue damage, promoting intestinal cell proliferation, and increasing the expression of TJ protein, ZO-1. Taken together, the findings indicated that the candidate peptide derived from the intestine may have protective effects on intestinal mucosal barrier function during IIRI.

To summarize, this research provides an in-depth evaluation of the process of IIRI from a peptidomics perspective. By comparing the peptidomics profiles in of the two groups (sham operated group and I/R group), this study systematically identified various DEPs involved in IIRI. Further, bioinformatics analyses revealed a close association between the peptide composition of the intestinal mucosal barrier and the pathogenesis of IIRI. In addition, this study identified a putative functional peptide, Actg2-6, which may prove to be a promising candidate for protection of the intestinal mucosal barrier against IIRI. However, this research is limited by the scope of the detection technology, the determined enzyme activity, and the number of samples. Nonetheless, the potential of the identified peptides as novel targets for therapy following IIRI still warrants in depth investigation. In addition, as this study only explored a small proportion of the peptides involved in intestinal mucosal barrier function and intestinal ischemia reperfusion, more work is required to uncover the possible molecular mechanisms of these peptides and their correlation with IIRI.
